# Family presence during resuscitation: A qualitative study of the experiences of families at the emergency medicine directorate of Komfo Anokye Teaching Hospital (KATH), Ghana

**DOI:** 10.1371/journal.pone.0307244

**Published:** 2026-01-02

**Authors:** Anokyewaa Boadi–Suadwa, Edward Appiah Boateng, Felix Apiribu, Cecilia Amponsem Boateng, Abena Kyerew Abebrese

**Affiliations:** 1 Department of Nursing, School of Nursing and Midwifery, Valley View University, Accra, Ghana; 2 Department of Nursing, School of Nursing and Midwifery, Kwame Nkrumah University of Science and Technology, Kumasi, Ghana; 3 Department of Nursing, School of Nursing and Midwifery, Kwame Nkrumah University of Science and Technology, Kumasi, Ghana; 4 Department of Nursing, School of Nursing and Midwifery, Valley View University, Accra Ghana; 5 Department of Nursing, School of Nursing and Midwifery, Kwame Nkrumah University of Science and Technology, Kumasi, Ghana; Universitair Kinderziekenhuis Koningin Fabiola: Hopital Universitaire des Enfants Reine Fabiola, BELGIUM

## Abstract

Family Presence During Resuscitation (FPDR) is increasingly recognized as a component of patient- and family-centred care, offering emotional support, fostering transparency, and strengthening trust in clinical care. However, in many Sub-Saharan African settings, including Ghana, the practice remains uncommon, undocumented, and lacks policy support. Fewer studies have examined families’ experiences with FPDR than those of clinicians. This study explored the lived experiences of Ghanaian families with witnessed resuscitation of their loved ones in a tertiary hospital in Ghana. A qualitative phenomenological approach was adopted to gain in-depth insights into the emotional, cognitive, and social experiences of family members present during resuscitation. Twelve participants were purposively sampled from the Emergency Medicine Directorate of Komfo Anokye Teaching Hospital (KATH). Data were collected through in-depth interviews and field notes and analyzed using Braun and Clarke’s Reflexive Thematic Analysis. Three major themes emerged: (1) Emotional Rollercoaster – families experienced shock, helplessness, and anxiety; (2) Uncertainty and Information Asymmetry – participants reported confusion and distress due to lack of communication and comprehension of information given; and (3) Decision Making and Consent – families encountered ethical dilemmas and emotional stress when making critical decisions under pressure. These findings reflect the core principles of patient- and family-centred care in the provision of dignity and respect, information sharing, and collaboration. It also highlights the cultural and emotional significance of FPDR in the Ghanaian context.

## 1. Introduction

### 1.1. Background and significance of the study

Resuscitation is a critical, life-saving medical intervention employed in emergencies such as cardiac arrest [1], severe trauma [2], or respiratory failure [3]. The series of life-saving activities employed to restore or improve cardiopulmonary function of lives are collectively known as Cardiopulmonary Resuscitation (CPR) [4]. It is often performed under high-stress conditions in emergency departments, where timely decisions and procedures determine patient survival outcomes [5]. Over the years, the concept of Family Presence During Resuscitation (FPDR), which allows family members to witness or remain present during resuscitation efforts, has gained traction as a patient- and family-centered care approach [6] in practice. FPDR is rooted in principles of transparency [7], emotional support and compassion [8], as well as informed decision-making [9].

Research in several high-income countries has shown that FPDR may facilitate closure and increase family trust in the healthcare system [10], and improve communication between clinicians and relatives [11]. Family members who witness resuscitation often feel reassured about the intensity and sincerity of the clinical efforts [12], which helps them process grief and accept outcomes [13]. Moreover, the presence of family during such procedures aligns with the broader movement toward humanizing emergency medicine and respecting patients’ social and emotional contexts [14].

Despite the growing global discourse, FPDR practice remains deeply contested and inconsistently implemented. Concerns from healthcare providers include fears of psychological trauma for the family [15], interference with clinical procedures [16], medico-legal consequences [17], and the absence of standardized protocols for managing family members during such critical moments [18]. These debates have led to a patchwork of policies across institutions and countries, with some advocating for formal FPDR programs [19] and others prohibiting it altogether [20].

### 1.2. Scientific and contextual gaps

Notably, in many low- and middle-income countries, including those in Sub-Saharan Africa, FPDR has received limited empirical attention despite similar or even greater needs for family involvement due to stronger kinship ties and communal caregiving norms [21]. In Ghana, there is no standardized policy or protocol for FPDR [22], and its practice is left to the discretion of clinicians or departments. Cultural expectations around respect for medical authority, religious beliefs, and social hierarchies further complicate family involvement in emergency care [23,24].

The scientific literature remains disproportionately centered on data from the Global North, primarily North America, Europe, Asia, and Australia [25–28], leaving a void in contextualized understanding of FPDR in African settings. This results in an evidence gap regarding how families in Ghana emotionally process FPDR, what support systems they require, and how clinicians navigate ethical, emotional, and practical challenges.

This study addresses these gaps by exploring, from a qualitative and phenomenological standpoint, the lived experiences of Ghanaian families who witnessed resuscitation efforts at a major tertiary hospital. By documenting emotional, cognitive, and social responses to FPDR, this study aims to generate localized, culturally-informed insights to guide policy and practice in Ghana and similar contexts.

### 1.3. Controversies and comparison of literature

While the benefits of FPDR have been documented in studies from the United States, Canada, and parts of Europe to enhance transparency and patient-family trust [29] controversies remain [30]. Critics argue that FPDR may heighten emotional distress [31], cause disruptive behavior during resuscitation [32], or result in litigation if outcomes are unfavorable [33]. Healthcare professionals fear losing control in emotionally charged situations and it is reported that while FPDR decreased anxiety in patients’ relatives, it simultaneously increased the stress of clinicians [34].

In contrast, literature from Sub-Saharan Africa is scarce. The few available studies in countries such as in Kenya [35,36], reveal the experiences of clinicians and less on family experiences. Nurses often lack formal training or institutional support to conduct FPDR, relying instead on personal discretion [35]. Family-centered care, although deeply valued in African cultural settings, remains operationally underdeveloped in emergency medicine across the region [37].

These regional differences highlight the consequences of unaddressed controversies. In high-resource settings, debates focus on balancing legal risks and ethical imperatives, whereas in low-resource settings, the very infrastructure to support or study FPDR is missing. This study, therefore, contributes to bridging this imbalance by providing context-specific evidence through the lenses of Ghanaian families, laying the groundwork for culturally competent emergency care frameworks.

## 2. Materials and methods

The Consolidated Criteria for Reporting Qualitative Research (COREQ) was used to report the methods and materials used in this study [38].

### 2.1. Research team and reflexivity

The research team comprised of five researchers with diverse backgrounds, including emergency medicine, social sciences and qualitative research. Team members had prior experience in qualitative data collection and analysis. To establish rapport with participants, the researchers were introduced to the participants by the clinical liaison for the team to develop trust. Efforts were made to minimize power dynamics by emphasizing the voluntary nature of participation. Journaling to record assumptions and biases done by researches in the course of data collection and analysis to ensure researcher reflexivity [39].

### 2.2. Study design and methodological justification

This is a qualitative study with a phenomenological approach [40]. This is well-suited for exploring the lived experiences of individuals in depth [41]. Phenomenology emphasizes understanding how individuals perceive and make meaning of a particular phenomenon, which in this case, is the experience of witnessing the resuscitation of a loved one [42]. Given the sensitive, complex, and deeply personal nature of Family Presence During Resuscitation (FPDR), this methodological approach allowed for a rich, nuanced understanding of participants’ emotional, cognitive, and social responses [43]. A purposive sampling technique [44]was employed to recruit participants with direct, lived experiences relevant to the research objective. This sampling strategy was appropriate because FPDR is a specific, situational, and not widely practiced in Ghana, requiring targeted recruitment [45] of individuals who had actually witnessed resuscitation events involving loved ones, to provide rich, contextually relevant insights into the FPDR experience. Again, given the sensitive nature and relatively rarity of the phenomenon in Ghanaian hospitals, random sampling was neither practical nor ethical.

The study included families who had visual contact during their loved ones’ resuscitation within the three months preceding the study’s initiation. Those unable to provide informed consent due to emotional distress or cognitive impairments were excluded. The research did not focus on specific medical conditions but encompassed any situation where witnessed resuscitation occurred. Out of the thirty-five participants approached, twenty agreed to participate in the study. Some declined due to ongoing grief, while others, whose relatives were still hospitalized, needed privacy during recovery. The researchers determined a sample size of twelve based on data saturation [46].

This was a hospital based study, which commenced on June 2023 at the Emergency Medicine Directorate of Komfo Anokye Teaching Hospital (KATH) in Ghana. The researchers selected this setting due to its appropriateness, as the Emergency Directorate is the largest in the northern part of Ghana and serves as the primary referral center for emergency cases, with an average daily admission of thirty emergency cases requiring resuscitation [47]. Non-verbal cues and contextual details were documented as field notes. Interviewers possessed experience in qualitative interviewing techniques and the ethical handling of sensitive topics, but researchers received additional training. Interviews were conducted in private consultation rooms within the emergency department to ensure confidentiality. All data were anonymized using pseudonyms. Participants who were unable to come to the hospital were interviewed in the privacy of their homes. The data collection period was three months, utilizing a semi-structured interview guide developed based on literature and expert input. Questions focused on participants’ physical and emotional responses to resuscitation, as well as cognitive and social experiences. Each interview lasted approximately 35–60 minutes and was audio-recorded with consent. To ensure the emotional and mental well-being of participants during the interview, participants were informed that they could request the termination of the interview at any point if they were unable to manage their emotions. Furthermore, all participants were independently assessed for emotional readiness by a mental health nurse before the commencement of the interview. Post-interview professional counseling was made available for participants on a referral basis.

### 2.3. Data analysis and findings

Thematic analysis was conducted using Braun and Clarke’s six-step framework [48] to identify, analyze, and interpret patterns in the data. NVivo 2020 was used to organize and code data systematically. Participant quotations were attached to illustrate themes. Afterward, the initial codes derived were independently reviewed by two of the researchers, and the discrepancies that came up were resolved through discussion among all researchers for peer validation and auditing. The emergent themes were reviewed and refined based on their alignment with the study objectives, and participant narratives have been clearly presented as results. An independent reviewer was engaged to ensure consistency and coherence. Recordings taken during interviews were saved on the password protected laptop of the principal investigator to be keep for a period of 5 years after which recordings may be disposed. In addition to this, recordings transcribed were compared with field notes to fill in gaps where clarity was needed.

### 2.4. Ethical approval and consent

The ethical approval for this study was obtained from the KATH Institutional Review Board (KATHIRB/AP/098/23) and the Committee on Human Research, Publications, and Ethics of Kwame Nkrumah University of Science and Technology (CHRPE/AP/1008/23).

Written consent was sought from participants after the purpose of the study was explained to them in a language that they clearly understand. Participant’s anonymity and confidentiality was maintained throughout the process of data collection. Each participant was fully informedout the study’s objectives, procedures, potential risks, and their right to withdraw at any point without repercussions. Audio recordings and transcripts were kept pass worded by the principal investigator.

## 3. Results

Twelve (12) participants were included in this study. Five (5) of the participants were females and seven (7) males. The age range of participants was thirty (30) to sixty-five (65) years. The relationship of participants to patients who received Cardiopulmonary Resuscitation (CPR) was made up of six (6) parental, five (5) spousal, and one (1) sibling. The demographic details of the participants are listed in Table 1.

**Table 1:** Demographic details of participants.

Pseudo Name	Participant’s Gender	Participant’s Age	Participant’s Occupation	Participant’s Religion	Participant’s Highest Qualification	Participant’s Relation to Patient	Family Member’s Diagnosis
P1	F	30	Trading	Christian	Junior High	Daughter of patient	Prostate Cancer
P2	F	41	Sales Executive	Christian	Tertiary	Daughter of patient	Severe Traumatic Brain Injury
P3	F	36	Waitress	Christian	Tertiary	Wife of patient	Septic Shock
P4	F	38	Hairstylist	Christian	Senior High	Wife of patient	Alcoholic Liver Disease
P5	M	50	Farming	Christian	Junior High	Husband of patient	Diabetic Ketoacidosis
P6	M	62	Tailoring	Christian	No Formal Education	Husband of patient	Breast Cancer
P7	M	55	Plumbing	Christian	No formal Education	Husband of patient	Hemorrhagic Stroke
P8	M	65	Unemployed	Islam	No Formal Education	Father of patient	Severe Traumatic
Brain Injury							
P9	M	56	Farming	Christian	Junior High	Father of patient	End Stage Kidney Disease
P10	M	48	Accountant	Christian	Tertiary	Son of patient	Hemorrhagic Stroke
P11	M	50	Trading	Christian	Senior High	Son of patient	Diabetic Ketoacidosis
P12	F	38	Teaching	Christian	Tertiary	Sister	Septic Shock

**Overview**: After data analysis, approximately 16 codes were generated from the transcripts. Table 2 depicts how the thematic development was done. Themes identified are 1. Emotional Rollercoaster, 2. Uncertainty and Information Asymmetry, 3. Decision Making and Consent. Themes, subthemes and codes are summarized in Table 2.

**Table 2:** Themes, subthemes, and codes.

Main Theme	Subtheme	Codes
1. Emotional Rollercoaster	a. Shock and Disbelief	a. Dissociative surreal
	b. Fear and Anxiety	b. Heightened Stress of the unknown
	c. Helplessness	c. Participatory Inability
2. Uncertainty and Information Asymmetry	a. Resuscitation Process Uncertainty	a. Non-familiarization
	b. Irregular updates on Resuscitation Activities	b. Restrictive information
	c. Update Comprehension Difficulty	c. Confusion/Brain Fog
3. Decision Making and Consent	a. Emergency Decision Stressors	a. Unrealistic choices
	b. Resuscitation Ethical Dilemmas	b. Morality

### 1. Emotional rollercoaster

The sudden and unexpected nature of resuscitation can intensify emotions and significantly impact families’ emotional well-being. The three subthemes that emerged were shock and disbelief, fear and anxiety, and helplessness.

***Shock and Disbelief***: Shock and disbelief were familiar emotional experiences expressed by the families who were interviewed. Shock was often characterized by a sense of feeling numb, disconnected, or detached from reality. In most cases, families who witnessed the resuscitation of their loved ones could not believe that such rigorous activities were necessary for their survival.

*“It felt like I was watching a movie, trying to appreciate what was happening to my wife” (P5).*

Families found it challenging to fully comprehend the situation and accept its severity. On the reverse, the shock response served as a protective mechanism, temporarily shielding families from the full emotional impact of the resuscitation event. As expressed by a participant below:

*“I didn’t feel anything in the beginning because I did not believe he was the one lying there. It was not until after a while that I felt my heart miss a beat. That was when I realized the real situation my son was in”,* (P9)

Disbelief, on the other hand, involved difficulty in accepting a situation. Some families experienced denial or disbelief, particularly when the sudden nature of the event did not align with their expectations or prior knowledge of the loved one’s health. This was expressed by a participant as:

*“I had just spoken to my husband over the phone while he was at work. In about 30 minutes, I was called by a friend that he had been rushed to the hospital. I just couldn’t believe the information until I got to the hospital and saw him, that was when I began shaking”,* (P4).

Both shock and disbelief were emotional responses that varied in intensity and duration from person to person. While it’s important to understand that these reactions were normal and part of the natural coping process, they persisted and significantly impaired some respondents’ ability to function over a period.

***Fear and Anxiety***: Fear and anxiety arose from the uncertainties surrounding the outcome of the resuscitation efforts. In other words, families were worried about the wellbeing and survival of their loved ones, and the fear of losing them was overwhelming. The fear of loss and the distressing nature of the resuscitation process itself can elicit strong emotional reactions. This is expressed by a participant below:

*“I feared the Doctors and Nurses would tell me that my mother didn’t survive despite all the efforts.”,* (P10).

Anxiety manifested as a feeling of unease, worry, or apprehension. From the study, families experienced physiological symptoms of anxiety, such as increased heart rate, difficulty in breathing, restlessness, and a sense of impending doom in some cases. These symptoms were distressing and overwhelming. This was described by a participant as:

*“I just kept pacing up and down the hallway. I felt my heart beating faster than usual. My palms were sweaty and I couldn’t hold back my tears”,* (P7).

Although fear and anxiety are expected emotional responses in challenging circumstances, if they become overwhelming, persistent, or significantly interfere with an individual’s daily functioning, they could have a long-lasting effect on their mental health.

***Helplessness:*** The study discovered that helplessness is another common emotion that families feel during the resuscitation process. Participants had wished to be involved in the resuscitation but found themselves unable to do so due to their lack of expertise. This feeling of helplessness intensified the emotional turmoil they were experiencing. This was expressed by a participant as follows:

*“At that moment, I wished I were God or an Angel, but there was nothing I could do except pray to Allah”,* (P8).

Witnessing a loved one receive emergency care evokes a sense of powerlessness and distress, as they often had limited control over the situation and the outcome of resuscitation efforts. A participant said this:

*“When I saw one Nurse cover him with the screen, I felt that was the end and my brother was dying”,* (P12).

### 2. Uncertainty and information asymmetry

Families experienced heightened uncertainty about whether their loved ones would survive or experience long-term complications.

***Resuscitation Process Uncertainty***: Generally, uncertainty is a significant source of distress in various aspects of life. In the context of resuscitation, families felt uncertain about the effectiveness of medical interventions, the response of their loved one’s body to the resuscitation activities, and the overall prognosis. This was described by a participant:

*“The doctor asked me to call someone who could bring the diabetic drugs he was taking, but, I could not even understand how it was going to help the situation, because, at that time, he could not even swallow anything”,* (P3)

There were times when the lack of precise answers created doubt in the minds of families. Therefore, the study showed that the lack of definitive answers and the unpredictable nature of the outcome can exacerbate these emotions.

***Irregular Updates on Resuscitation Activities***: Lack of information can be challenging for families during the resuscitation process. Understandably, families may be confused during such a critical and high-stakes situation. The study found that factors such as the complexity of medical procedures and lack of clear communication could lead to frustration during resuscitation because of a lack of regular updates.

In terms of the complexity of medical procedures, resuscitation procedures can be complex and may involve various medical interventions. Understanding the different steps involved, potential outcomes, and associated risks can be overwhelming for families encountering this situation for the first time. The lack of familiarity with medical terminology and processes can contribute to confusion. A participant said that:

*“I could hear the doctors and nurses talking to themselves, but I couldn’t not understand the big words they were saying to each other. I got more confused.”,* (P11).

Clear communication between clinicians and families is essential during the resuscitation process. The struggle to convey information in a clear and understandable manner is due to time constraints, high-stress environments in emergency wards, or a lack of specialized communication skills by clinicians. Consequently, families may feel uninformed about the decisions made regarding their relative. A participant said that:

*“But how could I keep calm when no one was telling me anything”,* (P9).

***Updates Comprehension Difficulty***: The emotional burden experienced by families during the resuscitation process can make it difficult for them to absorb and process the information provided by medical professionals, leading to further uncertainty and confusion. A participant said:

*“The Doctor explained to me what they were doing to get him to breathe again, but I was so confused and could not understand anything he said,”* (P8)

Study findings show that communication challenges during resuscitation can indeed be a significant concern for both healthcare providers and family members. While effective communication is vital, doctors and nurses may face certain hurdles in providing regular updates to anxious family members. Factors such as time constraints and emotional distress could lead to difficulty in explaining prognosis or understanding what information is shared by the Nurses or Doctors.

In terms of time constraints, resuscitation procedures often require immediate and focused attention from healthcare providers. In such situations, Doctors and Nurses may have limited time available to provide detailed updates to family members. Their primary focus is typically on the critical needs of the patient, which can make it challenging to communicate frequently or comprehensively. Another participant said:

*“The Doctor came out and pulled me aside and explained to me that they needed to put a tube in his mouth so that a machine would breathe for him. I could not get more details from him because he told me that he needed to be in the room to save my wife”, (*P5)

From the emotional distress perspective, the study findings show that family members awaiting updates during resuscitation may be experiencing heightened emotional distress and anxiety. These emotions can make it challenging for them to absorb information, and they may require additional support in understanding and processing the updates provided. A participant said that:

*“My mind was so far away that I could not bring myself to understand anything the Doctor was telling me,”* (P9).

### 3. Decision-making and consent

Findings from the study indicate that during resuscitation, medical professionals may indeed need to make quick decisions that can involve emergency procedures and potentially life-altering interventions. This is because resuscitation is typically performed in critical situations where immediate action is necessary to prevent further harm or save a person’s life. Decision-making in emergency settings in Ghana is shaped by a mix of traditional kinship systems, gender roles, respect for elders, religious beliefs, marital status and socioeconomic factors. While patriarchal norms dominate, the involvement of extended family, communal decision-making, and respect for cultural practices remain significant in these scenarios

***Emergency Decision Stressors***: The study found that making critical decisions during FPDR can be an incredibly stressful experience for families. Making important decisions under pressure can intensify stress. *A* participant said that:

*“The Doctor asked if I could get anyone I could call to donate blood for him urgently. This was in a highly stressful moment because no one came to mind immediately* (P4).

The stressors involved in making critical decisions during FPDR can arise from several factors, including the urgency of the situation, the emotional attachment to the patient, the complex medical information involved, and the fear of making the wrong decision. A participant said

*“The Doctor told me that I needed to give my consent for my son to have an emergency operation. I was so scared about how my son would survive a brain operation that I asked him to give me time to consider the options,”* (P8).

Findings further show that it is important to note that decision-making during resuscitation is guided by medical ethics principles, professional guidelines, and legal frameworks [49]. These guidelines promote the concept of shared decision-making when possible, involving the patient or their surrogate decision-maker in the process.

***Resuscitation Ethical Dilemma***: Findings from the study showed that families were faced with difficult decisions, such as whether to continue with resuscitation efforts, pursue further medical interventions, or consider alternative options such as palliative care. These decisions can have significant consequences and may require balancing potential benefits with potential harms or the quality of life for the loved one. A participant said that:

*“My father had battled with diabetes for so long that I agreed with the Doctors not to put him on a life support machine again but to let him go in peace if his heart stops beating again”,*

(P11).

In certain cases, medical professionals may need to perform resuscitation procedures without obtaining explicit consent from the patient or their family. This is considered an emergency medical intervention aimed at preserving life when consent cannot be obtained promptly. However, medical professionals are expected to respect the principle of autonomy and make efforts to involve the patient or their family in the decision-making process when feasible. A participant said:

*“In fact at this point, the Doctor made me aware that the liver had failed and the only solution was to get him a new one. He said the operation for this is expensive and it is not even done in Ghana. Besides, we do not even know if someone in our family will donate a liver to him. So I agreed not for the resuscitation to continue if his heart stopped beating. Besides, he is so sick that even if we have the money and a liver for him, he may not survive the operation.”,* (P4)

Findings also reveal that time is often of the essence in resuscitation scenarios, as delays in initiating certain interventions may significantly impact the patient’s chances of survival. Medical professionals must make decisions swiftly and efficiently based on their training, experience, and available information.

## 4. Discussion

This study sought to explore the lived experiences of Ghanaian families who were present during the resuscitation of their loved ones, with the aim of deepening understanding of Family Presence During Resuscitation (FPDR) within the Ghanaian emergency healthcare context. The findings are congruent with core principles of patient-centred care (PCC) and family-centred care (FCC), both of which emphasize dignity, respect, shared decision-making, emotional support, and involvement of families in care [50].

The emotional rollercoaster experienced by families, including shock, helplessness, anxiety, and grief, demonstrates the profound psychological and relational dimensions of healthcare [51]. When families are present during these critical moments, it affirms the principle of respect for persons, a foundation of PCC that acknowledges the right of individuals and their families to be informed and involved in the care process. FPDR also facilitates transparency and trust, as witnessing the intensity of care can validate the clinical team’s effort and reduce perceptions of negligence, a significant concern in healthcare interactions in Sub-Saharan Africa [52].

In line with FCC principles, FPDR recognizes the family as integral to the patient’s support system. Families expressed a desire not only to be present but to be included in the care narrative [53]. This reflects broader cultural norms in Ghana, where communal values and kinship obligations shape the way health crises are managed. Therefore, FPDR is not only a clinical issue but a culturally embedded one.

Clinically, the findings highlight the importance of communication and emotional support during resuscitation. The resuscitation process itself can be intense and may involve a race against time, which further adds to family emotional distress. Families who received timely and comprehensible updates, even amid chaos, experienced a greater sense of inclusion and emotional stability [53]. Training clinicians to engage in empathetic, concise communication, even during critical procedures, is a key recommendation. Furthermore, having dedicated personnel to support families during resuscitation could improve clinical outcomes by allowing clinicians to focus while families receive the reassurance and clarity they need.

Although scholarly work on this is limited, the second theme which came out as uncertainty and information asymmetry resonated with the participants. The uncertainty of resuscitation and its outcomes, coupled with inadequate information and regular updates on resuscitation, increased the frustration of families during FPDR. Furthermore, the study highlighted how families’ inability to fully comprehend resuscitation activities while present can be unsettling. This was evident in how some families who lacked an understanding of medical procedures and the complexities of resuscitation, coupled with a stressful environment, found themselves experiencing heightened emotional stress.

Providing regular updates to families can be stressful for emergency clinicians because it may distract them from their main focus of resuscitating the patient [54]. This finding is corroborated by this study, which found regular updates and explanations of resuscitation activities to families by clinicians, though stressful on clinicians, can be an effective way to manage the difficult experience of witnessing the resuscitation of a loved one. This is an indication for clinicians to prioritize clear and compassionate communication, using plain language, to help families understand the resuscitation process, potential outcomes, and available options.

Decision Making and Consent highlights the third theme which brings to light the daunting challenge of families making difficult decisions within a limited time frame. This was a common experience among participants. The added pressure of making critical decisions on behalf of their incapacitated loved one within a limited timeframe, heightens emotional turmoil, making families grappled with overwhelming fear, anxiety, and grief [55]. The intense emotional state of families witnessing resuscitation can lead to cognitive overload, impeding their ability to think rationally and process medical information. The compressed timeframe amplifies the burden of decision-making, potentially leading to impaired judgment and increased stress. Ethically, clinicians have the burden of guiding family members to make decisions in the best interest of the patient during times when patients are unable to communicate their will, such as patients undergoing resuscitation [56].

Healthcare professionals must provide clear and concise explanations of available options and potential outcomes, though they may face the challenge of time constraints. Transparent communication empowers families to navigate the decision-making process more effectively. This is significant when families face the challenging decision to discontinue resuscitation efforts and transition to alternative treatments such as palliative care, recommended by the United States Best Practice Guidelines for Primary Palliative Care in the Emergency Department [57]. The delicate balance between hope and realistic expectations makes this decision emotionally and ethically burdensome for both clinicians and families.

### 4.1. Policy and clinical implications

This study offers critical insights that should inform policy formulation and clinical practice:

a. Policy Development: The Ghana Health Service and hospital administrations should prioritize the creation of FPDR protocols aligned with international best practices, customized for cultural relevance.
b. Workforce Training: Emergency staff should receive training on how to handle FPDR scenarios, including trauma-informed communication and ethical decision-making.
c. Infrastructure Support: Emergency departments should be equipped with private viewing spaces and staffing models that allow for simultaneous patient care and family support.
d. Post-Resuscitation Debriefing: Institutions should implement debriefing mechanisms to help families process the experience and receive emotional or spiritual support.

These implications are particularly relevant in strengthening patient safety, dignity, and family trust in Ghanaian healthcare.

### 4.2. Limitations and potential solutions

While this study contributes significantly to understanding FPDR in Ghana, certain limitations must be acknowledged:

a. Small Sample Size and Scope: The study involved twelve participants from a single tertiary hospital, which may limit generalizability.

*Solution*: Future research could expand the sample across multiple regions and healthcare institutions for broader representation.

b. Recall Bias: Since participants recounted emotionally intense events retrospectively, some details may have been unintentionally distorted.

*Solution*: Future studies could consider immediate post-event interviews where appropriate, or longitudinal follow-up to assess the lasting impact.

c. Lack of Clinician Perspectives: This study focused solely on families; insights from emergency clinicians could provide a balanced understanding of FPDR.

*Solution*: A mixed-methods approach or parallel studies involving clinicians would enrich the findings.

d. Absence of Policy Audit: The study did not examine existing institutional policies or lack thereof on FPDR at the facility level.

*Solution*: Future studies could include a review of institutional policies and readiness for FPDR implementation.

## 5. Conclusion

This study provides valuable evidence on the experiences of Ghanaian families who witnessed the resuscitation of their loved ones. It situates FPDR within the broader frameworks of patient-family-centred care, emphasizing its potential to humanize care, foster trust, and uphold ethical principles of autonomy and shared decision-making. In the Ghanaian context, where cultural, familial, and communal bonds are deeply embedded in the healthcare journey, FPDR represents not only a clinical strategy but a culturally congruent practice that deserves institutional support.

As a low-resource setting striving toward equitable and compassionate healthcare, Ghana must consider the policy integration and clinical operationalization of FPDR as part of its broader health systems strengthening agenda. Future research to explore formalization of FPDR through training, infrastructure development, and evidence-based policies that prioritize both the patient and their families during some of the most vulnerable moments in care is required.

### 5.1. Summary

**What is known about the topic**

i FPDR is not a regular practice in the emergency departments in Ghana
ii Clinicians are reluctant to practice FPDR, due to legal concerns and the destructive nature of attending to families during resuscitation.

**What this Study Adds**

i The perspective of Ghanaian families with the concept of FPDR in patient- family centered care
ii During FPDR families experience emotional outbursts for which clinicians need to be educated to be accommodating and sensitive to such family needs.
iii Regular communication and updates in clear and simple language can help families cope with the outcome of resuscitation
iv Families give consent to difficult decisions willingly when they can comprehend the activities of resuscitation.

## Supporting information

S1 Data Transcribed Data. (DOCX)
