# Supplementary material for: Family presence during resuscitation: A qualitative study of the experiences of families at the emergency medicine directorate of Komfo Anokye Teaching Hospital (KATH), Ghana
Source: PLoS One. 2026 Jan 2;21(1):e0307244. doi: 10.1371/journal.pone.0307244 (PMC12758757; doi:10.1371/journal.pone.0307244)
Supplement: S1 Data — (DOCX) [file pone.0307244.s001.docx]

**SECTION A – TRANSCRIBED RESPONSES ON THE INTERVIEW GUIDE**

**Physical Responses**

Participant 1:

"My hands were shaking so badly, I couldn’t even hold onto my phone. My legs felt weak like I might collapse any second, but I couldn’t leave—I had to stay."

Participant 4:

"There was this tightness in my chest like I couldn’t breathe properly. The noise in the room—machines beeping, people talking—made it even worse. It felt like my body was in fight-or-flight mode the whole time."

Participant 3:

"I couldn’t feel anything for a while. It was like my body had gone numb, but then I realized I was gripping the chair so hard that my fingers hurt."

Participant 6:

"The bright lights and constant movement around me made it hard to focus. It was overwhelming, like sensory overload—I couldn’t process everything happening at once."

**Emotional Responses**

Participant 5:

"At first, I felt paralyzed by fear. But then I saw the doctor nod, and I thought, ‘Maybe there’s a chance.’ That moment gave me hope, but it didn’t last long. When they started talking about time, I knew it wasn’t good."

Participant 2:

"I was heartbroken but also angry—angry at the situation, angry that this was happening. I didn’t know where to direct all that emotion, so I just stood there crying."

Participant 11:

"I felt this overwhelming sadness, like I was already mourning, but I couldn’t fully let go because they were still trying. It was this strange mix of grief and hope that I couldn’t escape from."

Participant 10:

"After it was over, I felt guilty for even hoping. It’s like my hope had betrayed me, and I should’ve been more realistic. That guilt stayed with me for days."

**Cognitive Responses**

Participant 9:

"I kept thinking, ‘What is that machine for?’ or ‘Why are they pressing so hard on his chest?’ I had no idea if they were making progress or if it was already too late."

Participant 4:

"I wanted to ask questions, but I felt like I’d be in the way. I was so focused on trying to understand what was happening, but it all felt so complicated and fast."

Participant 7:

"I remember wondering if they had missed something—like, was there something more they could have done earlier? Those thoughts haunted me long after the event."

Participant 12:

"The whole thing made me question modern medicine. They were doing so much, but it didn’t seem to help. It made me wonder if we rely too much on technology and not enough on knowing when to stop."

**Social and Cultural Experiences**

Participant 8:

"No one really spoke to me during the resuscitation. A nurse came over after to say they were sorry, but by then, it felt too late. I needed reassurance while it was happening, not after."

Participant 4:

"I was there alone because no one else could make it in time. I think being by myself made it worse—I didn’t have anyone to hold onto or talk to about what I was seeing."

Participant 5:

"The doctor explained what they were doing, but it was all medical jargon. I felt like they were trying, but I still didn’t really understand. It made me feel small, like I wasn’t part of the process."

Participant 6:

"Afterwards, my sister blamed me for letting them keep going for so long. It’s created this rift between us because we see the situation so differently—I wanted to fight for him, and she thinks I should have told them to stop sooner."

**Overall Reflections**

Participant 7:

"Looking back, I think being there helped me. At least I know they tried everything, even if it didn’t work. But it’s something I relive in my mind constantly—it’s not an easy memory to carry."

Participant 8:

"I’m torn about it. On one hand, I’m glad I was there so he wasn’t alone. But on the other hand, I think it added to my trauma. Sometimes I wish I hadn’t seen it."

Participant 9:

"I think family members should have the option to be there, but only if they’re fully prepared. I wasn’t prepared—I didn’t know what I was walking into, and that made it harder."

Participant 4:

"If I could go back, I’d ask more questions. I was so caught up in the moment that I didn’t advocate for myself. I think if I had understood more, it wouldn’t haunt me as much."

**SECTION B – TRANSCRIBED RESPONSES EXTRAPULATED INTO THEMES**

**Theme 1: Emotional Roller Coaster**

Participant 1:

"When they started chest compressions, I felt a surge of panic. My heart was racing, and I kept hoping they’d save him, but when the monitor flatlined, I just collapsed inside. It was hope, fear, and then just devastation."

Participant 2:

"I was so scared, but then I’d see them working so hard, and for a moment, I’d think, ‘Maybe he’ll make it.’ That feeling kept swinging back and forth—hope to hopelessness."

Participant 3:

"The emotions were like a storm—I was crying one moment and then frozen the next. After it was over, I felt so empty and drained, like I’d been on a roller coaster I didn’t ask to ride."

Participant 10:

"At first, I was numb—it was like I wasn’t really there. But then I heard the monitor beep faster, and my heart jumped. For a second, I thought it was a good sign, but when it slowed again, I just started sobbing. It was this constant back-and-forth of hope and despair."

Participant 11:

"I remember feeling anger—anger at the situation, anger that it was happening, and even anger at myself for not knowing what to do. Then guilt took over, like maybe I could have done something to prevent it. My emotions didn’t make sense; they were all over the place."

Participant 12:

"It was like waves crashing over me. When they started using the defibrillator, I thought, ‘This will work.’ But when nothing changed, I felt like I was drowning. Even now, I still get choked up thinking about it."

**Theme 2: Irregular Updates on Resuscitation Activity**

Participant 4:

"I kept looking at the doctors, but no one was telling me what was happening. Was it good? Was it bad? I just stood there, not knowing what to think. It made the whole thing so much harder."

Participant 5:

"There was this long stretch where I had no clue what they were doing. I was just watching and guessing—every movement felt like it was life or death, but no one explained anything."

Participant 6:

"One nurse would say, ‘We’re doing everything we can,’ and then nothing for 10 minutes. It felt like hours. The waiting without any updates was unbearable—I just needed to know, even if it was bad news."

Participant 7:

"I stood there, just staring at their faces, trying to read them for clues. Were they worried? Was it working? I didn’t know if I should ask or stay quiet—I didn’t want to interrupt, but the silence was unbearable."

Participant 1:

"One of the nurses came over briefly and said, ‘We’re still trying,’ but that was it. It felt so vague. I kept waiting for someone to tell me something concrete, like, ‘This is what’s happening now,’ but no one did."

Participant 9:

"I kept seeing them write things down or look at monitors, and I just wanted to scream, ‘What does that mean?!’ I think if someone had just explained what they were doing step by step, I wouldn’t have felt so helpless."

Participant 2:

"The most frustrating part was not knowing how long it would last. I kept thinking, ‘Is this normal? Should I prepare for the worst?’ The communication gaps left me stuck in this awful limbo."

**Theme 3: Ethical Dilemma**

Participant 12:

"At one point, I wondered if they should stop. He’d been through so much already, and I wasn’t sure if we were just prolonging his suffering. But then again, how could I say that? It felt so wrong to even think it."

Participant 8:

"Watching them work so hard, I kept thinking, ‘Is this what he would’ve wanted?’ It’s hard to know if I should’ve said something or just trusted them to do what’s best."

Participant 9:

"I felt torn—part of me wanted them to keep going, to do everything they could, but another part of me wondered if we were just putting him through more pain for nothing. It felt like an impossible decision."

Participant 1:

"He had told me before that he didn’t want to be kept alive if things got really bad. But when I saw them trying so hard, I couldn’t bring myself to say anything. I felt like I was betraying him by not speaking up, but I also didn’t want to be the one to decide."

Participant 3:

"I kept wondering if they were doing it for him or for us. Was it their job to keep trying no matter what? Or were they hoping we’d step in and say, ‘That’s enough’? I didn’t know what the right thing to do was."

Participant 9:

"I felt like I was caught between two responsibilities—letting him go peacefully or fighting for him to stay alive. I kept thinking, ‘Am I being selfish for wanting him to stay? Or would it be selfish to let him go?’ It was an impossible choice."

Participant 2:

"At one point, I started questioning the doctors. Were they pushing too far? Should I have said something? But then I thought, ‘What if they stop and he could have made it?’ It’s something I still wrestle with."

**How These Responses Strengthen the Themes:**

Emotional Roller Coaster is reinforced by participants’ intense and shifting feelings of fear, hope, anger, sadness, and guilt. The lack of clear updates amplified the emotional roller coaster, as participants swung between hope and despair without understanding what was happening.

Irregular Updates on Resuscitation Activity become more pronounced as participants reflect on the silence, vague communication, or technical jargon from the medical team.

The ethical Dilemma is further clarified through participants questioning the appropriateness of resuscitation, their roles in decision-making, and the moral conflict of wanting to let go versus holding on.

**Summary of Themes**

Emotional Roller Coaster: Participants described fluctuating emotions of fear, hope, sadness, and devastation, often overwhelming and persisting after the event.

Irregular Updates on Resuscitation Activity: A lack of clear, timely communication during the resuscitation heightened participants’ stress, confusion, and feelings of helplessness.

Ethical Dilemma: Participants reflected on internal conflicts regarding the appropriateness of continuing resuscitation and whether it aligned with the patient’s wishes or overall well-being
